# World Facing the latest variant of Concern---------Omicron

**DOI:** 10.4314/ejhs.v32i2.29

**Published:** 2022-03

**Authors:** Muhammad Mohsin Javaid, Ayesha Fazal, Mawra Hyder, Farooq Ahmad Chaudhary, Shahab Ud Din

**Affiliations:** 1 Demonstrator, Community & Preventive Dentistry Department, School of Dentistry, Shaheed Zulfiqar Ali Bhutto Medical University /PIMS, Islamabad, Pakistan; 2 Demonstrator, Community & Preventive Dentistry Department, School of Dentistry (SOD), Shaheed Zulfiqar Ali Bhutto Medical University/PIMS, Islamabad, Pakistan; 3 Demonstrator, Community & Preventive Dentistry Department, School of Dentistry (SOD), Shaheed Zulfiqar Ali Bhutto Medical University/PIMS, Islamabad, Pakistan; 4 Assistant Professor, Community & Preventive Dentistry Department, School of Dentistry (SOD), Shaheed Zulfiqar Ali Bhutto Medical University/PIMS, Islamabad, Pakistan; 5 Professor, Department of Science of Dental Materials, School of Dentistry (SOD), Shaheed Zulfiqar Ali Bhutto Medical University/PIMS, Islamabad, Pakistan

**TO THE EDITOR**: On November 24, 2021, from the Republic of South Africa, “B.1.1.529” variant was revealed and conveyed to WHO. The first confirmed and verified B.1.1.529 infection was from the specimen which was collected on November 9, 2021 (1). On the advice of “Technical Advisory Group on Virus Evolution” (TAGVE) for WHO , this variant was termed as “Omicron” on November 26, 2021, (2). So far, 363 Million cases and 5.63 Million deaths have been reported world widely due to COVID-19. As of 20^th^ January 2022, the Omicron variant had been identified in 171 nations across all 6 WHO Regions (3).

A novel VOC, the Omicron variant (Pango lineage B.1.1.529 and sublineages BA.1 and BA.2), Omicron S protein evades antibodies with up to 44-fold higher efficiency than the spike of the Delta variant, providing therapeutic antibodies that are ineffective and likely to compromise protection by antibodies induced upon infection or vaccination with two doses of BNT162b2 (BNT).

**Additional amino acid changes monitored were** +S:R346K (4).

Scientists and researchers across the world are now rushing to understand the threat Omicron has been imposing. One of the major worries regarding omicron is the uncertainty of the coverage of COVID vaccine against this variant. Until now, more than thirty mutations of this variant have been documented. These mutations lead to higher antibody escape, enhanced viral binding affinity and increased transmissibility. With the aim to protect and guard against this newly noticed variant, vaccines manufacturers for the COVID-19 are thoughtful regarding the modification of their products (5,6). The term “variant of concern” (VOC) for SARS-CoV-2 (which produces COVID-19) refers to viral variants with mutations in their spike protein receptor-binding domain (RBD) that dramatically improves binding affinity in the RBD-hACE2 complex while also causing fast dissemination in human populations (7).

Observing epitope variability has applications in disease monitoring, diagnostic settings, and drug design. A mutation occurring on the specific epitope range may affect the recognition of the epitope. Vast interest has been showed on studying the epitopes, parts of SARS-CoV-2 sequence which can be recognized by drugs, vaccines, as well as serological tests. For epitopes, “Immune Epitope Database” (IEDB) is known as the utmost important, fully public repository, as of to date collecting approximately 5000 epitopes of the SARS-CoV-2 (along with several other viruses), well-described by the means of search panels and attributes. Numerous computational tools are also used for supporting the forecast of epitopes. EpiSurf-system covers a different need, as per it provides a flexible interface for testing their conservancy, measured as the absence/presence of amino acid variations/changes over the sequences of epitope (8).

Some reports have revealed that pathogensity and affinity of Omicron have been reduced. One study validates that Omicron variant is attenuated in the virus replication and pathogenicity in comparison to the other previous variants. One more research found that, Omicron variant is lesser effective in the replicating cells. Replication of live Omicron was reduced as related to original strain and other variants of SARS-CoV-2. They documented that Omicron was more than 3 times lesser efficient at the viral multiplication in comparison to that of original COVID19 strain in the lung epithelial cells of humans. In contrast, Alpha, Beta & Delta variants replicated at almost similar or higher levels than original virus (9).

Individuals who are inoculated are anticipated to have a lesser threat of serious ailment from this infection. Approach and policy of getting inoculation at governmental level and public health protective measures (wearing of masks, physical distancing, preference of open-air gathering, avoidance of fenced spaces, and preservation of hand hygiene) are anticipated as an effective strategy (10).

Several countries had banned air journeys from Omicron suffered countries and Governments are also administering the booster doses. States are strictly enforcing the vaccination regime. Avoidance of crowding. Nations are also making sure that the populace is wearing mask at the public places. Hand Sanitizers are at the entrance of public places. Ban on in door gatherings has been emphasized

Cases of Omicron has surfaced in Pakistan as well. When compared with other countries, Pakistan has low vaccination rate. So, it can possibly be dangerous for the unvaccinated populace of Pakistan, once again overloading the healthcare system. Thus, it is imperative to take concrete and tangible measures to halt the spread of this variant. Genome sequencing centers are the need of the hour and should be rapidly developed across the nation (11).

According to latest reports, a consistent upsurge has been observed across Europe and U.S.A including U.K, France etc. Folks with previous inoculations and booster dosages will not be affected as such, populace with waned vaccinations are more at risk (12).

South African experts have listed fatigue, mild fever, scratchy throat, night sweats, body ache and dry cough to be the symptoms seen in Omicron patients. Sense of smell and taste are no longer affected in patients, making it harder for the person affected to realize the virus's presence (13).

According to recent studies, it has been detected that no vaccines have proved successful in stopping the new variant from spreading but the effects recorded are mild as compared to the previous variants. Currently tests are being conducted on blood samples taken from Omicron patients to understand the effects as well as how the vaccine reacts to the new variant (14).
